# The cycad genotoxin methylazoxymethanol, linked to Guam ALS/PDC, induces transcriptional mutagenesis

**DOI:** 10.1186/s40478-024-01725-y

**Published:** 2024-02-21

**Authors:** Bert M. Verheijen, Claire Chung, Ben Thompson, Hyunjin Kim, Asa Nakahara, Jasper J. Anink, James D. Mills, NYGC ALS Consortium, Hemali Phatnani, Justin Kwan, Dhruv Sareen, James R. Broach, Zachary Simmons, Ximena Arcila-Londono, Edward B. Lee, Vivianna M. Van Deerlin, Neil A. Shneider, Ernest Fraenkel, Lyle W. Ostrow, Frank Baas, Noah Zaitlen, James D. Berry, Andrea Malaspina, Pietro Fratta, Gregory A. Cox, Leslie M. Thompson, Steve Finkbeiner, Efthimios Dardiotis, Timothy M. Miller, Siddharthan Chandran, Suvankar Pal, Eran Hornstein, Daniel J. MacGowan, Terry Heiman-Patterson, Molly G. Hammell, Nikolaos A. Patsopoulos, Oleg Butovsky, Joshua Dubnau, Avindra Nath, Robert Bowser, Matthew Harms, Eleonora Aronica, Mary Poss, Jennifer Phillips-Cremins, John Crary, Nazem Atassi, Dale J. Lange, Darius J. Adams, Leonidas Stefanis, Marc Gotkine, Robert H. Baloh, Suma Babu, Towfique Raj, Sabrina Paganoni, Ophir Shalem, Colin Smith, Bin Zhang, Brent Harris, University of Maryland Brain and Tissue Bank and NIH NeuroBioBank, Iris Broce, Vivian Drory, John Ravits, Corey McMillan, Vilas Menon, Lani Wu, Steven Altschuler, Yossef Lerner, Rita Sattler, Kendall Van Keuren-Jensen, Orit Rozenblatt-Rosen, Kerstin Lindblad-Toh, Katharine Nicholson, Peter Gregersen, Jeong-Ho Lee, Maze Therapeutics, Bristol-Myers Squibb, Sulev Koks, Stephen Muljo, Bryan J. Traynor, Pfizer, Regeneron, Insitro, Jeong H. Lee, Eleonora Aronica, Kiyomitsu Oyanagi, Akiyoshi Kakita, Jean-Francois Gout, Marc Vermulst

**Affiliations:** 1 School of Gerontology, University of Southern California, Los Angeles, CA 90089 USA; 2 Department of Neuroscience, Maastricht University, Maastricht, The Netherlands; 3 Graduate School of Medical Science and Engineering, Korea Advanced Institute of Science and Technology (KAIST), Daejeon, Republic of Korea; 4 Department of Neurology, Asan Medical Center, University of Ulsan College of Medicine, Seoul, Republic of Korea; 5 Department of Pathology, Brain Research Institute, Niigata University, Niigata, Japan; 6 Department of Neuropathology, Amsterdam UMC, University of Amsterdam, Amsterdam Neuroscience, Amsterdam, The Netherlands; 7 Department of Clinical and Experimental Epilepsy, University College London Queen Square Institute of Neurology, London, WC1N 3BG UK; 8 Chalfont Centre for Epilepsy, Chalfont St Peter, SL9 0RJ UK; 9 Division of Neuropathology, Department of Brain Disease Research, Shinshu University School of Medicine, Matsumoto, Nagano Japan; 10 Department of Biological Sciences, Mississippi State University, Mississippi State, MS 39762 USA

**Keywords:** Guam amyotrophic lateral sclerosis/parkinsonism–dementia complex, Environmental toxin, DNA damage, O^6^-mG, Transcriptional mutagenesis

Guam amyotrophic lateral sclerosis/parkinsonism–dementia complex (ALS/PDC) is a rare neurodegenerative disorder with high prevalence among the native Chamorro population of Guam (Mariana Islands) [1–3]. ALS/PDC presents clinically as progressive motor neuron disease (resembling classic ALS), parkinsonism with dementia, or a combination of both. At the neuropathological level, ALS/PDC is characterized by tau-dominant multiple proteinopathy (Fig. 1) (an overview of neuropathology can be found in [4]). The etiology of ALS/PDC is unclear, although involvement of both genetic and environmental factors has been suggested. The prevalence of ALS/PDC has declined dramatically in Guam, coincident with rapid westernization [5, 6]. Additionally, migration studies indicate that disease risk is increased after prolonged residence in the geographic cluster [7]. These findings hint at an important role for environmental or lifestyle factors in the disease. Recent whole-genome sequencing (WGS) analysis of postmortem brain and spinal cord tissues from ALS/PDC cases did not find evidence for neurogenetic causes (Additional File 1) and results of cryogenic electron microscopy analysis of tau filaments from ALS/PDC cases are in line with an environmental etiologic hypothesis [8]. There is great interest in understanding the cause of ALS/PDC in Guam, because insight into its origins may also yield clues as to the cause of common neurodegenerative diseases throughout the world [9, 10].

**Fig. 1:**
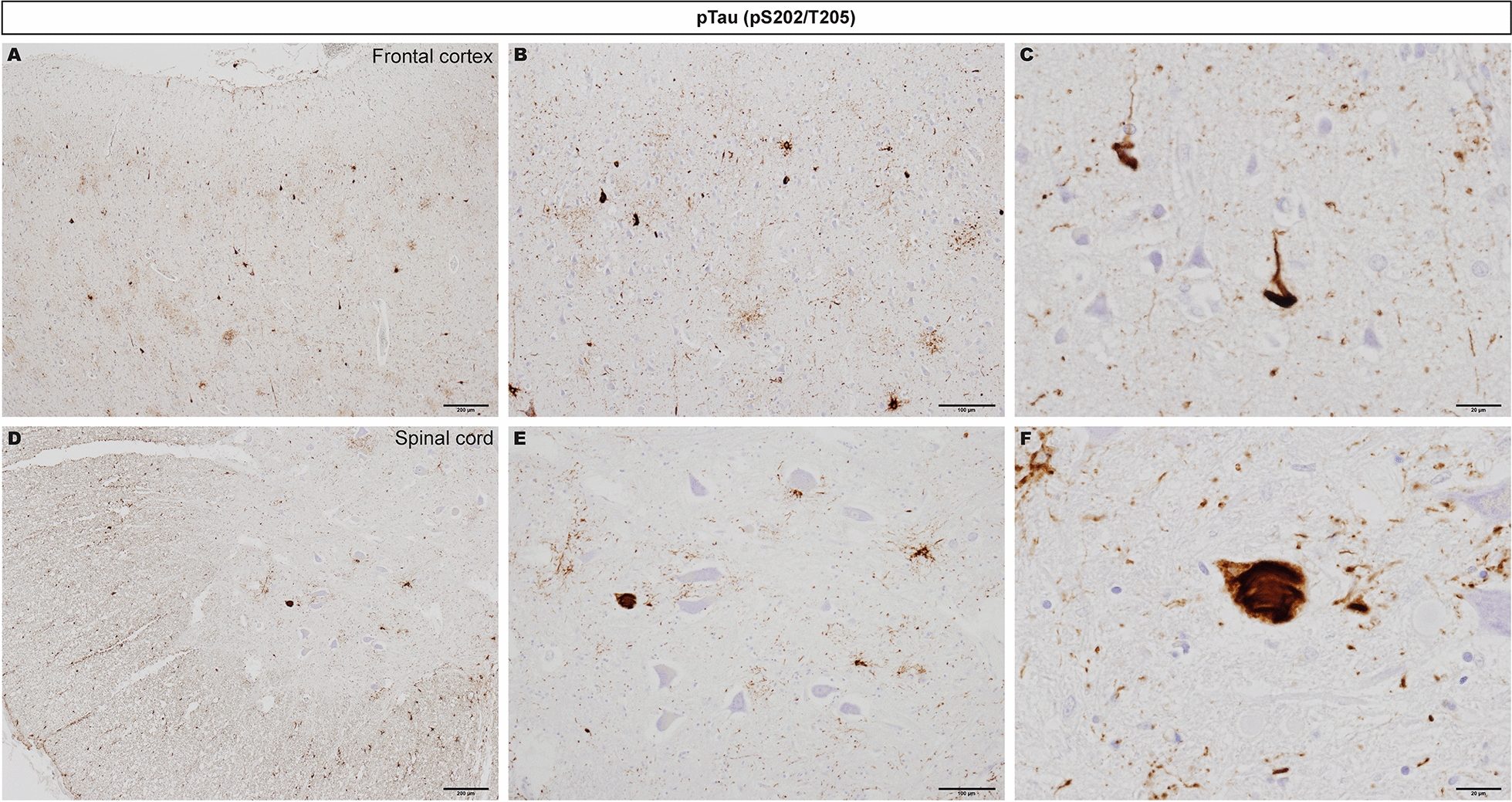
Phosphorylated tau (pTau) aggregates in Guam ALS/PDC tissues. pTau cytoplasmic inclusions in brain (**A**–**C**) and anterior horn of the spinal cord (**D**-**F**) in Guam ALS/PDC cases (AT8, Innogenetics). **A**–**C** and **D–F** show increasing magnifications of representative immunostained tissue sections. pTau aggregates in brain and spinal cord are well-known pathological hallmarks of ALS/PDC and were used for diagnosis of cases included in Additional File 1. Tissue sections correspond to Guam PDC subject #3 (**A–C**) and #1 (**D–F**) from [8]

One hypothesis for the cause of ALS/PDC in Guam involves exposure to toxins present in cycad plants. Epidemiological work has shown a significant association between exposure to cycad and neurological disease on Guam [11]. Cycad seeds are known to contain several potentially toxic agents, e.g., cycasin and its aglycone methylazoxymethanol (MAM) [12, 13] and β-N-methylamino-L-alanine (BMAA) [14, 15], which have both been tenuously linked to neurological disease in Guam. The cycad-associated toxin MAM is a potent DNA alkylating agent that has been shown to induce O^6^-methylguanine (O^6^-mG) lesions in DNA [13]. Previous work has found that such DNA lesions in particular are associated with a process known as transcriptional mutagenesis (TM) that introduces mutations, not present in DNA, into newly synthesized RNA molecules [16]. Errors in transcription have been linked to proteotoxic phenotypes [17], and may play a role in the pathogenesis of ALS/PDC [18, 19]. Although the cycad hypothesis for ALS/PDC has fallen into disfavor [20], it remains unknown whether MAM exposure promotes TM.

To determine the effects of MAM on TM, we used a single-cell RNA-sequencing (scRNA-seq) approach. We argued that if a lesion is present at a specific site in DNA, it could result in the generation of a substantial number of “misread” transcripts that carry an RNA–DNA discrepancy at that same specific location. If transcripts are tagged with a unique molecular identifier (UMI), it should be possible to perform sequence comparisons of transcripts present in a single cell. Such comparisons would not be feasible using bulk RNA-sequencing, because reads in bulk RNA-seq data may correspond to distinct DNA lesions in different cells. This scRNA-seq strategy has recently been used in another study [21], in which arrested yeast cells and quiescent mouse neural stem cells (NSCs) were exposed to *N*-methyl-*N*′-nitro-*N*-nitrosoguanidine (MNNG), a chemical compound that is routinely used to induce O^6^-mG DNA lesions and RNA synthesis errors [16, 22], and subsequently used as input material for scRNA-seq. It was found that certain transcription errors do indeed occur repetitively as a consequence of MNNG exposure. Additionally, those experiments showed that exposure to MNNG induced expression of genes associated with protein quality control machinery [21]. For the experiments with MAM, we treated a homogeneous culture of quiescent NSCs with 1 mM MAM acetate or vehicle (phosphate-buffered saline [PBS]) for 1 h. We used NSCs, because these are neural cells (i.e., they represent a disease-relevant cell type) and because they can be easily put in a quiescent state by altering the composition of the culturing medium (Fig. 2A). Using quiescent cells was important, to prevent fixation of DNA damage into DNA mutations, which would confound transcription error measurements (this is also why non-replicating yeast cells and quiescent NSCs were used in previous experiments with MNNG). Next, cells were rinsed with PBS to remove MAM from the culturing medium, after which they were allowed to recover in quiescence medium for 16 h. Again, an important consideration is that, as long as DNA damage is not repaired, bursts of transcription on the damaged DNA will result in mutant RNAs that contain an error at the same location as the corresponding damage site. After the incubation period, cells were collected for scRNA-seq experiments. The single-cell data demonstrated that MAM-treated NSCs display an increase in C → U errors (average error rate: 6.9 × 10^–5^ bp^−1^) as compared to control (vehicle-treated) cells (average error rate: 6.7 × 10^–6^ bp^−1^) (Fig. 2B) (Additional File 2). C → U errors are the primary type of transcription error induced by O^6^-mG lesions [16] and are consistent with MAM-induced DNA damage. Importantly, there was no comparable increase in G → A errors in MAM-treated samples, which shows that mutations in DNA do not underlie this effect. Interestingly, we could identify erroneous transcripts that were present in a ≥ 50:50 ratio to WT transcript (Fig. 2C). This suggests that certain DNA lesions can mimic the effect of a DNA mutation. In separate experiments, rolling-circle consensus sequencing (CirSeq) of RNA isolated from MAM-treated vs. vehicle-treated primary mouse fibroblasts indicated an increased overall transcription error output (N → N) following MAM treatment (Additional File 2).

**Fig. 2:**
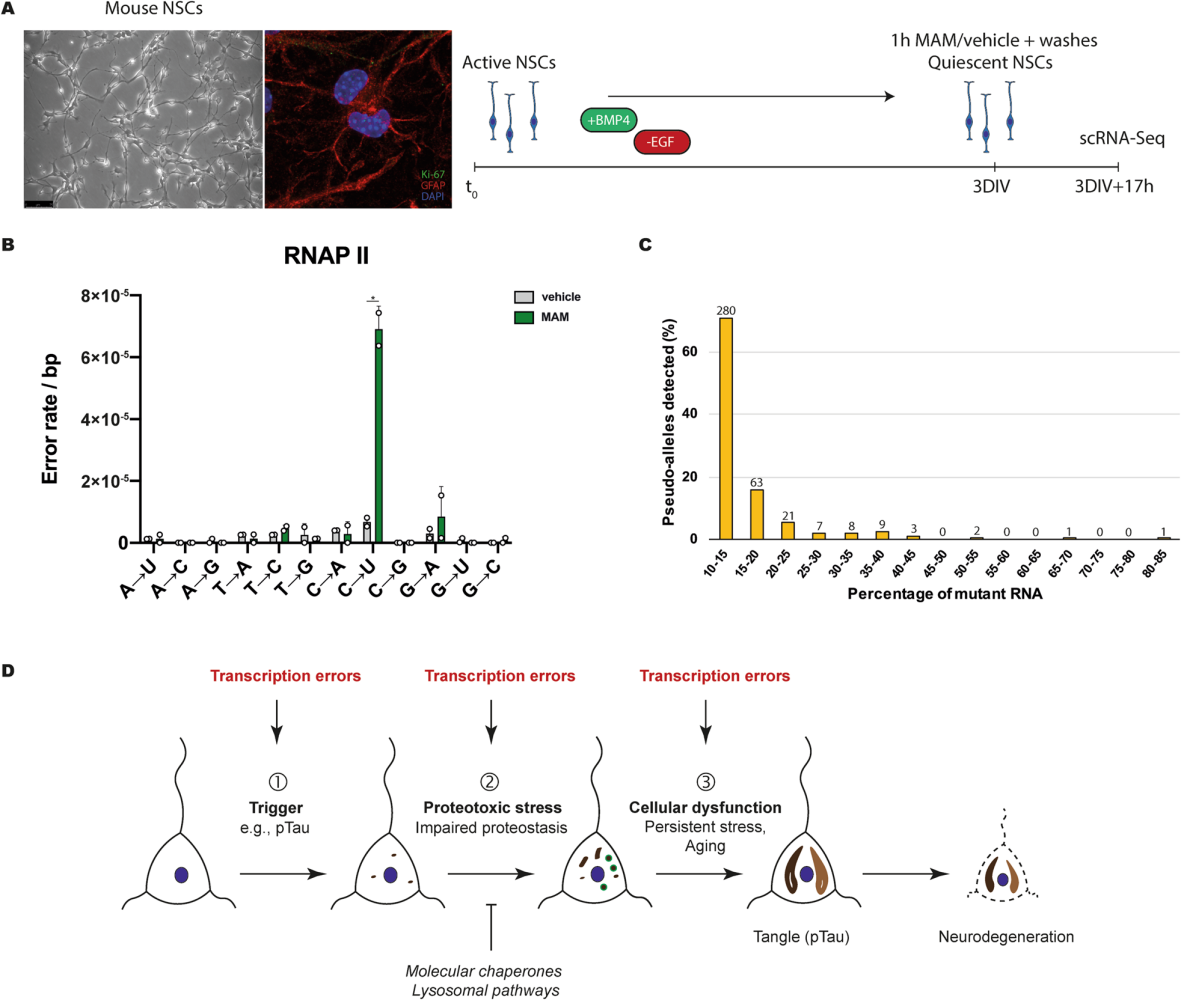
Methylazoxymethanol (MAM) exposure induces transcriptional mutagenesis in neural stem cells. **A** Overview of the experiment. Mouse primary hippocampal neural stem cells (NSCs) were cultured in quiescence medium for 3 days. Cell cycle arrest was validated by Ki-67 staining. Next, quiescent NSCs were treated with 1 mM methylazoxymethanol (MAM) acetate or vehicle (PBS) for 1 h, after which cells were rinsed with PBS and cultured for 16 more hours in quiescence medium. Cells were then collected and used for single-cell RNA-seq experiments. **B** The error spectrum of RNAPII in MAM-treated NSCs shows an increased C → U error rate as compared to vehicle-treated cells. *P < 0.01, unpaired two-tailed t-test. **C** MAM treatment results in transcripts containing identical errors, termed pseudo-alleles for their ability to generate both WT and mutant transcripts. The graph depicts the ratio of mutant:WT mRNAs detected. Only alleles with more than 10% mutant mRNAs are included (MAM-treated replicate #1). **D** It is proposed that (1) mutant RNAs, which are the result of transcriptional mutagenesis on unrepaired O^6^-methylguanine (O^6^-mG) DNA lesions, could initiate disease by generating toxic molecules, e.g., misfolded proteins that act as proteopathic seeds. Additionally, (2) an overall increase in the number of erroneous RNAs could overwhelm the cellular protein quality control machinery, potentiating proteotoxic stress phenotypes by impairing the clearance of toxic proteins. Lastly, (3) transcription errors could promote the transition of stressed cells to a dysfunctional state through various mechanisms

This data demonstrates that exposure to MAM, an environmental genotoxin, induces TM. The full biological significance of faulty RNAs generated through TM remains to be determined. Previous work has shown that an overall increase in transcription errors causes a profound loss of proteostasis and potentiates the toxicity of disease-related proteins in cells [17]. Additionally, these errors could contribute to the generation of the amyloid proteins that drive neurodegeneration [23] (Fig. 2D). Even though the cause of ALS/PDC remains unknown, and cycad toxins are an unlikely culprit [20], it will be interesting to explore the links between DNA damage, transcript errors, protein misfolding and aggregation phenotypes in more detail in future work. These findings may also have implications for the study of other environmental toxins (e.g., pesticides) and their relation to neurological disease as well as other disorders like cancer. The in vitro experiments with MAM described here should ideally be complemented by other assays, such as DNA damage and mutation detection assays, and should preferably also be extended to human cells (e.g., human induced neurons) and intact animals. We anticipate that transcript error analysis will be a valuable addition to (neuro)toxicology testing.

### Supplementary Information

**Additional file 1.** Supplementary material 1 (ZIP 1015 kb)

**Additional file 2.** Supplementary material 2 (ZIP 444 kb)

**Additional file 3.** Supplementary Table 1. NYGC ALS Consortium members (XLSX 14 kb)

## Data Availability

The accession number for the scRNA-seq datasets reported in this paper is BioProject: PRJNA988009. The accession number for the CirSeq datasets reported in this paper is BioProject: PRJNA1054124.
