# Supplementary material for: The cycad genotoxin methylazoxymethanol, linked to Guam ALS/PDC, induces transcriptional mutagenesis
Source: Acta Neuropathol Commun. 2024 Feb 21;12:30. doi: 10.1186/s40478-024-01725-y (PMC10882831; doi:10.1186/s40478-024-01725-y)
Supplement: Supplementary file 1 — Additional file 1. Supplementary material 1 (ZIP 1015 kb) [file 40478_2024_1725_MOESM1_ESM.zip › Additional File 1/Supplementary Information 1.pdf]

Supplementary Information 1

Whole-genome sequencing and RNA-sequencing analysis of Guam ALS/PDC postmortem brain and spinal cord tissues

Bert M. Verheijen, Claire Chung, Ben Thompson, Hyunjin Kim, Asa Nakahara, Jasper J. Anink, James D. Mills, NYGC ALS consortium, Jeong H. Lee, Eleonora Aronica, Kiyomitsu Oyanagi, Akiyoshi Kakita, Jean-Francois Gout, Marc Vermulst

Frozen brain (frontal cortex) and spinal cord tissues from Guam amyotrophic lateral sclerosis/parkinsonism–dementia complex (ALS/PDC) cases and matched Chamorro controls were used for the whole-genome sequencing (WGS) and RNA-seq experiments described here. An overview of selected tissue samples available at the start of the study (n=45) can be found in **Supplementary Table 2**.

Whole-genome sequencing analysis of Guam ALS/PDC nervous tissues

The discovery on the frequent occurrence of ALS/PDC in a geographically isolated population, combined with the familial nature of the disease, initially suggested a genetic etiology. However, no Mendelian pattern of inheritance has been demonstrated (1). Previous work has shown that variation in the microtubule-associated protein tau gene (*MAPT*) contributes to disease risk, but does not cause ALS/PDC (2, 3). Additional loci on chromosome 12 have been suggested (4), however no single gene has been causally linked to ALS/PDC of Guam.

Here, WGS analysis was performed on postmortem nervous tissues to explore a role of pathological germline variants in ALS/PDC. A description of the ALS/PDC cases (n=14) and controls (n=9) included for WGS is provided in **Supplementary Table 3** (metadata associated with samples included for WGS analysis can be found in **Supplementary Table 4**).

We first checked the WGS data for a number of common disease-associated mutations. A GGGGCC (G_4C_2) hexanucleotide repeat expansion in the chromosome 9 open reading frame 72 gene (*C9ORF72)* is the most common genetic cause of ALS and frontotemporal dementia (5, 6) and partly accounts for ALS in the Kii peninsula of Japan (7), which prompted us to investigate *C9ORF72* repeat status in Guam ALS/PDC. In agreement with previous work (8), pathogenic *C9ORF72* repeat expansions (> 24 G_4C_2 repeats) were not found to be present in the evaluated Guamanian cases (**Supplementary Table 3**). Concordantly, C9 dipeptide-repeat (DPR) proteins (i.e., poly-GA, poly-GR), generated by unconventional repeat-associated translation of C9ORF72 transcripts (9), were absent from ALS/PDC brain (frontal cortex and hippocampus) and spinal cord tissues on immunohistochemistry (Verheijen et al., unpublished data). Intermediate CAG repeat expansions (30 – 33 CAG repeats) in the ataxin-2 gene (*ATXN2*) on chromosome 12, recognized as a risk factor for ALS (10, 11), were also not detected in any of the Guamanian tissues analyzed (**Supplementary Table 3**). The ε4 allele of the apolipoprotein E gene (*APOE*) is the strongest genetic risk factor for Alzheimer's disease and has also been shown to increase risk for other neurological disorders (12-14). However, *APOE* alleles appear to be mostly neutral (ε3) in Guam ALS/PDC (**Supplementary Table 3**), in keeping with previous findings in Guamanians (3, 15, 16). Twenty of the 23 individuals evaluated had two copies of the common ε3 allele, with two ALS/PDC cases having a ε3/ε4 allele, and one control having a ε3/ε2 allele. *APOE* status was suggested to modify tau pathology in Kii ALS/PDC (17), but because *APOE* alleles were mostly neutral in the Guamanian cohort we could not explore this link in the present study. Guam ALS/PDC has previously been linked to loci related to the *MAPT* gene. Using three of these possible disease-linked loci (rs242557, rs2258689, and rs242944) (3) we examined the genotypes of individuals. Again, we observed no discernible difference between the ALS/PDC cases and healthy controls (HCs) (rs242557, A/A, ALS/PDC: 6/14 = 42.8%, HC: 3/9 = 33.3%; rs2258689, C/C,

ALS/PDC: 0/7 = 0%, HC: 0/9 = 0%; rs242944, G/-, ALS/PDC: 9/9 = 100%, HC: 6/6= 100%) (**Supplementary Table 3**).

Next, the HaplotypeCaller variant-calling algorithm was used to seek for germline variants (18). To generate variant datasets containing only the variants with the highest confidence, a stringent filtering process was performed, including exclusion of known single nucleotide variants (SNVs) from the dbSNP database (19-21). In the case of filtered SNVs, additional filtering criteria were applied to identify potentially pathogenic mutations, including Ensembl Variant Effect Predictor (VEP) impact as high or moderate, combined annotation dependent depletion (CADD) score > 20, and minor allele frequency ≤ 0.05% (22, 23). These filtered lists of SNVs and indels were evaluated for variants previously associated with ALS (24). Two SNVs and three indels were identified as ALS-related gene variants. We found no evidence that these variants increase genetic risk for disease (**Supplementary Table 5**).

These results do not support a simple genetic cause for ALS/PDC of Guam. However, we cannot rule out a complex genetic etiology or a role for gene-environment interactions in ALS/PDC. Possible (low-level) post-zygotic or mitochondrial mutations could not be examined in this data (in addition, pathogenic splice mutations cannot be identified by WGS alone, but would require paired DNA- and RNA-seq analysis). A recent report including WGS analysis of Guam ALS/PDC brains also did not find evidence for increased genetic risk of neurodegenerative disease phenotypes (25).

In addition to scanning the WGS data for potentially pathogenic genetic variants, an analysis of mutational signatures (from the Catalogue of Somatic Mutations in Cancer [COSMIC]) was performed (26). This analysis may recognize specific mutational processes that were active in the evaluated tissues, and allowed us to investigate the possibility of a distinct germline mutational signature presenting within the ALS/PDC cases compared to the controls. Examination of single base substitutions showed that SBS1 (a clock-like signature associated with spontaneous deamination of 5-methylcytosine), SBS5 (a clock-like signature of unknown etiology), and SBS16 (unknown etiology) were dominant in both Guam ALS/PDC cases and Guam controls (**Sup. Fig. 1**). These signatures can be mainly attributed to aging. Similar mutational signatures were observed in neurons in previous work using a duplex sequencing protocol (27).

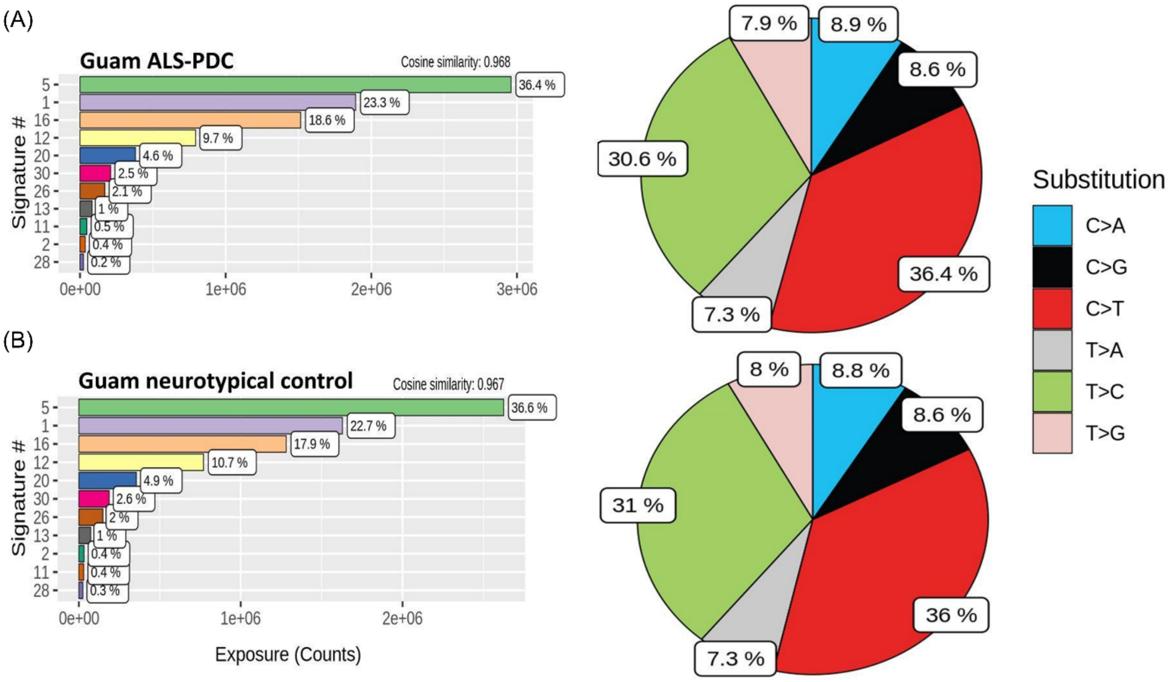

Sup. Fig. 1. Mutational signatures in Guam ALS/PDC cases and Guam controls. Analysis of single base substitution (SBS) mutational signatures (COSMIC) in Guam ALS/PDC cases (A) and Chamorro controls (B) shows that SBS1 (a clock-like signature associated with spontaneous deamination of 5-methylcytosine), SBS5 (a clock-like signature of unknown etiology) and SBS16 (unknown etiology) are the dominant signatures in both datasets. These mutation signatures can be mainly attributed to aging.

RNA-sequencing analysis of Guam ALS/PDC nervous tissues

RNA was isolated from frozen brain (frontal cortex) and spinal cord tissues and used for RNA-sequencing experiments. Metadata associated with samples included for RNA-seq analysis (including RNA integrity number [RIN]) can be found in **Supplementary Table 6.**

Principal component analysis (PCA) of all samples (n=31) showed clear segregation of tissue types (brain vs. spinal cord) (**Sup. Fig. 2A**). Two samples were excluded from further analysis, because tissue type designation did not match sequencing data (as indicated in PCA plot) (**Sup. Fig. 2A**).

Samples were allocated to different groups based on tissue type (brain; spinal cord), sex (male; female) and disease state (ALS; PDC; ALS/PDC; control) for gene expression comparisons. Groups with sample size n=1 were excluded from analysis. Differential gene expression analysis (DESeq2) of disease cases vs. controls did not identify many differentially expressed genes (FC > 2, FDR < 0.1) and most differentially expressed transcripts identified were not shared between groups (**Supplementary Table 7**). In the female PDC cortex vs. control cortex comparison, a number of inflammation-associated genes was found to be significantly up-regulated, e.g., *complement component 3 (C3)* (FC 2.51) and *triggering receptor expressed on myeloid cells 2 (TREM2)* (FC 3.80) (**Sup. Fig. 2B**) (**Supplementary Table 7**). These expression differences indicate active neuroinflammatory processes in PDC brains, e.g., through involvement of activated microglia (28, 29). Indeed, neuroinflammation has been linked to Guam ALS/PDC neuropathological change in previous work (30, 31), and may act as an important driver of disease progression. Therefore, these expression differences are biologically plausible. Whether neuroinflammation represents a *bona fide* biological pathway altered in the majority of ALS/PDC cases remains to be determined in more detail. *C3* and *TREM2* expression levels were not altered in any of the other group comparisons (**Supplementary Table 7**).

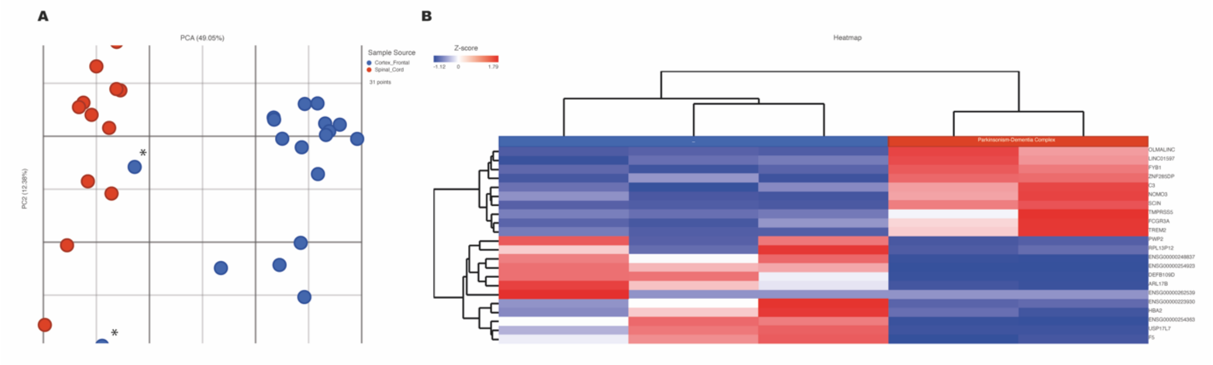

Sup. Fig. 2. RNA-seq analysis of Guam ALS/PDC and Chamorro control brain and spinal cord tissues. (A) Principal component analysis (PCA) plot of RNA-seq data (n=31) shows separation of tissue types, i.e., frontal cortex vs. spinal cord. Two samples (marked with an *asterisk*) did not match *a priori* tissue type designation and were excluded from further analysis. (B) Differential gene expression analysis of female Guam PDC cortex (n=2) vs. female Chamorro control cortex (n=3) identifies few significantly altered transcripts (FC > 2, FDR < 0.1), as shown in this heatmap.

Limitations

Several limitations should be noted, which could account for the absence of consistent differential gene expression patterns. First, the overall number of cases included was rather low, which resulted in limited statistical power. Additionally, heterogeneity among samples could explain a lack of significant expression changes. The included samples could represent different disease stages in which different disease processes are active. Furthermore, the heterogeneity of the disease itself should be considered. Lack of a common genetic predisposing factor could also contribute to the absence of shared expression changes. Moreover, there may have been substantial differences in tissue sampling. For example, the finding on differential expression of *oligodendrocyte maturation-associated long intergenic non-coding RNA* (*OLMALINC)* (**Supplementary Table 7**), a long non-coding RNA that is specifically expressed in white matter (32), indicates tissue collection from different brain regions, perhaps with different degrees of degeneration, resulting in a dilution effect. Microarray analysis of Kii ALS/PDC postmortem tissues primarily recognized stress-response pathways associated with pathological changes as being altered, which are mostly localized processes in individual cells (33). Single-cell RNA-sequencing (scRNA-seq) and spatial transcriptomics approaches could provide more insight into localized gene expression changes in ALS/PDC nervous tissue. Expression changes related to oxygen and iron homeostasis (*hemoglobin subunit beta* [*HBB]*, *hemoglobin subunit alpha 2* [*HBA2]*) were shared between some of the Guam ALS/PDC samples (**Supplementary Table 7**), and were also present in previously published data on Kii ALS/PDC (33), but it is not certain whether this reflected actual biological change or if it was caused by blood contamination of the tissue samples. Importantly, it is not clear whether Chamorro controls are the proper controls for these experiments. Previous studies have found that neurofibrillary tangles (NFTs) are also frequently present in postmortem brains of Guamanians without overt neurological disease (34, 35). We did not detect NFTs in a limited number of Guam control cases (n=3) in a previous study, but the mean age of these controls (mean age: 46.7 years) was considerably lower than the mean age of disease cases (mean age: 58.8 years) (36) (please note that average age of controls was stated as 47.7 years in the original paper as to not underestimate age due to rounding). Due to the limited availability of tissues, it was not feasible to include more Guamanian samples in our experiments, but additional non-neurological control cases from non-Chamorro backgrounds should be used for comparisons in future studies.

Methods

Whole-genome sequencing

DNA was extracted from frozen nervous tissues (frontal cortex or spinal cord) and processed for sequencing by the NYGC ALS Consortium (https://www.nygenome.org/als-consortium/). All samples underwent rigorous quality assessment using a comprehensive set of quality measures upon completion of each step of sample processing (i.e., sample receipt, library preparation, sequencing, and data analysis).

First, DNA was extracted from tissues using the QIAamp DNA Mini Kit (QIAamp #51306) according to the manufacturer's recommendations. DNA samples were quantified using fluorescent-based assays (PicoGreen or Qubit) to accurately determine whether sufficient material was available for library preparation and sequencing. DNA sample size distributions were profiled by a Fragment Analyzer (Advanced Analytics) or BioAnalyzer (Agilent Technologies), to assess sample quality and integrity. DNA was separately aliquoted for SNP array genotyping to determine DNA integrity and identity ahead of sequencing. These genotyping results were also checked for gross sample contamination and could reveal other forms of poor sample quality prior to sequencing.

WGS libraries were prepared using either the Illumina TruSeq PCR-Free Library Preparation Kit (n=23) or the Illumina TruSeq Nano DNA Library Preparation Kit (n=2) (as specified in **Supplementary Table 4**). Each whole genome library was prepared by following the manufacturer's instructions. Briefly, for TruSeq PCR-Free libraries, 1 μg of DNA was sheared using a Covaris LE220 sonicator (Adaptive Focused Acoustics). DNA fragments underwent end-repair, bead-based size selection, adenylation, and Illumina sequencing adapter ligation. For TruSeq Nano DNA libraries, 100 ng of DNA was sheared using the Covaris LE220 sonicator. DNA fragments underwent end-repair, bead-based size selection, adenylation, and Illumina sequencing adapter ligation. Ligated DNA libraries were enriched with PCR amplification (using 8 cycles). PicoGreen was used to measure the total amount of DNA in the prepared libraries. To measure the amount of adapter-ligated DNA (ligation efficiency) that is compatible with sequencing, quantitative PCR (qPCR) was used with specific oligos complimentary to Illumina's TruSeq adapters. Size distribution profiles of the final libraries were assessed using the Fragment Analyzer/BioAnalyzer.

Paired-end sequencing was performed on an Illumina NovaSeq 6000 sequencer using 2x150bp cycles (NovaSeq V1.5). All sequencing runs were reviewed for quality (library cluster efficiency/% Pass Filter (PF) clusters; % sample de-multiplexed; # of PF reads/sample; % bases > Q30; quality by cycle; GC content; k-mer content/adapter contamination).

Sequencing reads were aligned to the GRCh38 human reference using the Burrows-Wheeler Aligner (BWA-MEM v0.7.15) to perform a number of preliminary analyses, e.g., gender concordance (by comparing the gender information obtained from the sequencing to the gender specified at sample submission), ancestry analysis (an estimate of individual genome-wide average ancestries from a set of SNP genotypes using the ADMIXTURE tool

[http://dalexander.github.io/admixture/publications.html], which is a maximum likelihood-based method), and repeat expansion analysis of *ATXN2* and *C9ORF72* (call of repeat expansions in PCR-free WGS data using ExpansionHunter v2.5.5) (37). Results of those analyses are summarized in **Supplementary Table 4**.

Next, a separate WGS analysis was performed to explore additional aspects of the data. We followed the GATK best practices workflow to generate analysis-ready BAM files. Briefly, the FASTQ files were aligned to the GRCh38 human reference genome assembly via Burrows-Wheeler Alignment. Duplications within the data were marked and base quality scores recalibrated for all raw mapped reads in BAM format, resulting in analysis-ready BAM files. HaplotypeCaller (18) was used to call potential germline SNV and indels within each BAM file, resulting in VCF (Variant Call Format). In order to produce variant datasets consisting of only high-confidence calls, the following filtering criteria were introduced: QUAL > 30, QD > 10, mapping quality (MQ) > 30 and allelic depth (AD) > 10. Variants previously observed within the healthy population were removed also, by excluding known SNPs in the dbSNP database and indels within the 1000 Genome database (19-21). Using the VEP annotation algorithm, VCF files could be analyzed based on the likelihood of their impact being deleterious. SNVs were therefore carried forward only if their VEP impact was that of "HIGH" or "MODERATE", CADD score > 20, and minor allele frequency ≤ 0.05% (22, 23). For mutational signature analysis, variants that passed the filtering criteria (QUAL, QD, MQ, AD) were used as input into a single-base substitution signature analysis script (38).

Bulk RNA-sequencing

RNA was isolated from frozen nervous tissues (frontal cortex or spinal cord) and processed for sequencing by the NYGC ALS Consortium (https://www.nygenome.org/als-consortium/). Briefly, total RNA was isolated from tissues using Trizol-chloroform extraction, followed by Qiagen RNeasy minikit column purification. RNA was quantified using fluorescent-based assays (RiboGreen or Qubit) and purity of the extracted RNA was measured by Nanodrop 2000 analysis (A260/280 ratio). RNA sample size distributions were profiled by a Fragment Analyzer (Advanced Analytics) or BioAnalyzer (Agilent Technologies) to assess sample quality and integrity.

Libraries were prepared using the KAPA Stranded RNA-Seq Kit with RiboErase (KAPA Biosystems) in accordance with the manufacturer's instructions. Briefly, 500 ng of total RNA was used for ribosomal depletion and fragmentation. Next, depleted RNA underwent first and second strand cDNA synthesis. cDNA was then adenylated, ligated to Illumina sequencing adapters, and amplified with 9 cycles of PCR for sequencing on the NovaSeq 6000 platform (NovaSeq V1). PicoGreen or a Qubit Fluorometer was used to measure the total amount of prepared library. Library size distribution profiles of the final libraries were assessed using the Fragment analyzer/Bioanalyzer.

All RNA-seq data analysis was performed using Partek Flow software (Partek). Briefly, sequencing reads (associated with 31 samples, including both Guam ALS/PDC cases and Chamorro controls) were trimmed based on quality score (input parameters - trim based on:

quality score; min read length: 25; end min quality level [Phred]: 20; trim from end: 3-prime [right end]; quality encoding: autodetect). Trimmed reads were aligned to the GRCh38 reference genome using STAR aligner (39) and data was quantified to the Partek E/M annotation model. Additionally, the htseq-count script from HTSeq (40) was used for quantifying aligned reads. Next, gene counts were filtered (filter features, exclude features where: maximum ≤ 10.0) and normalized (median ratio DEseq2). Normalized counts were used for DEG analysis (DESeq2) (41) on different groups (split attributes - tissue type; sex; disease state).

References

1. Morris HR, Steele JC, Crook R, Wavrant-De Vrieze F, Onstead-Cardinale L, Gwinn-Hardy K, et al. Genome-wide analysis of the parkinsonism-dementia complex of Guam. Arch Neurol. 2004;61(12):1889-97.
2. Poorkaj P, Tsuang D, Wijsman E, Steinbart E, Garruto RM, Craig UK, et al. TAU as a susceptibility gene for amyotropic lateral sclerosis–parkinsonism dementia complex of Guam. Arch Neurol. 2001;58(11):1871-187811708997.
3. Sundar PD, Yu CE, Sieh W, Steinbart E, Garruto RM, Oyanagi K, et al. Two sites in the MAPT region confer genetic risk for Guam ALS/PDC and dementia. Hum Mol Genet. 2007;16(3):295-306.
4. Sieh W, Choi Y, Chapman NH, Craig UK, Steinbart EJ, Rothstein JH, et al. Identification of novel susceptibility loci for Guam neurodegenerative disease: challenges of genome scans in genetic isolates. Hum Mol Genet. 2009;18(19):3725-38.
5. DeJesus-Hernandez M, Mackenzie IR, Boeve BF, Boxer AL, Baker M, Rutherford NJ, et al. Expanded GGGGCC hexanucleotide repeat in noncoding region of C9ORF72 causes chromosome 9p-linked FTD and ALS. Neuron. 2011;72(2):245-56.
6. Renton AE, Majounie E, Waite A, Simon-Sanchez J, Rollinson S, Gibbs JR, et al. A hexanucleotide repeat expansion in C9ORF72 is the cause of chromosome 9p21-linked ALS-FTD. Neuron. 2011;72(2):257-68.
7. Ishiura H, Takahashi Y, Mitsui J, Yoshida S, Kihira T, Kokubo Y, et al. C9ORF72 repeat expansion in amyotrophic lateral sclerosis in the Kii peninsula of Japan. Arch Neurol. 2012;69(9):1154-8.
8. Dombroski BA, Galasko DR, Mata IF, Zabetian CP, Craig UK, Garruto RM, et al. C9orf72 hexanucleotide repeat expansion and Guam amyotrophic lateral sclerosis-Parkinsonism-dementia complex. JAMA Neurol. 2013;70(6):742-5.
9. Mackenzie IR, Frick P, Neumann M. The neuropathology associated with repeat expansions in the C9ORF72 gene. Acta Neuropathol. 2014;127(3):347-57.
10. Elden AC, Kim HJ, Hart MP, Chen-Plotkin AS, Johnson BS, Fang X, et al. Ataxin-2 intermediate-length polyglutamine expansions are associated with increased risk for ALS. Nature. 2010;466(7310):1069-75.
11. Glass JD, Dewan R, Ding J, Gibbs JR, Dalgard C, Keagle PJ, et al. ATXN2 intermediate expansions in amyotrophic lateral sclerosis. Brain. 2022;145(8):2671-6.
12. Feringa FM, van der Kant R. Cholesterol and Alzheimer's Disease; From Risk Genes to Pathological Effects. Front Aging Neurosci. 2021;13:690372.

13. Raulin AC, Doss SV, Trottier ZA, Ikezu TC, Bu G, Liu CC. ApoE in Alzheimer's disease: pathophysiology and therapeutic strategies. Mol Neurodegener. 2022;17(1):72.

14. Fernandez-Calle R, Konings SC, Frontinan-Rubio J, Garcia-Revilla J, Camprubi-Ferrer L, Svensson M, et al. APOE in the bullseye of neurodegenerative diseases: impact of the APOE genotype in Alzheimer's disease pathology and brain diseases. Mol Neurodegener. 2022;17(1):62.

15. Buee L, Perez-Tur J, Leveugle B, Buee-Scherrer V, Mufson EJ, Loerzel AJ, et al. Apolipoprotein E in Guamanian amyotrophic lateral sclerosis/parkinsonism-dementia complex: genotype analysis and relationships to neuropathological changes. Acta Neuropathol. 1996;91(3):247-53.

16. Galasko D, Salmon D, Gamst A, Olichney J, Thal LJ, Silbert L, et al. Prevalence of dementia in Chamorros on Guam: relationship to age, gender, education, and APOE. Neurology. 2007;68(21):1772-81.

17. Sasaki R, Morimoto S, Ozawa F, Okano H, Yoshida M, Ishiura H, et al. APOE Alleles With Tau and Abeta Pathology in Patients With Amyotrophic Lateral Sclerosis and Parkinsonism-Dementia Complex in the Kii Peninsula. Neurology. 2022;99(22):e2437-e42.

18. Poplin R, Ruano-Rubio V, DePristo MA, Fennell TJ, Carneiro MO, Van der Auwera GA, et al. Scaling accurate genetic variant discovery to tens of thousands of samples. Biorxiv. 2018.

19. Sherry ST, Ward MH, Kholodov M, Baker J, Phan L, Smigielski EM, et al. dbSNP: the NCBI database of genetic variation. Nucleic Acids Res. 2001;29(1):308-11.

20. Fairley S, Lowy-Gallego E, Perry E, Flicek P. The International Genome Sample Resource (IGSR) collection of open human genomic variation resources. Nucleic Acids Res. 2020;48(D1):D941-D7.

21. Mills RE, Luttig CT, Larkins CE, Beauchamp A, Tsui C, Pittard WS, et al. An initial map of insertion and deletion (INDEL) variation in the human genome. Genome Res. 2006;16(9):1182-90.

22. McLaren W, Gil L, Hunt SE, Riat HS, Ritchie GR, Thormann A, et al. The Ensembl Variant Effect Predictor. Genome Biol. 2016;17(1):122.

23. Kircher M, Witten DM, Jain P, O'Roak BJ, Cooper GM, Shendure J. A general framework for estimating the relative pathogenicity of human genetic variants. Nat Genet. 2014;46(3):310-5.

24. Iacoangeli A, Al Khleifat A, Sproviero W, Shatunov A, Jones AR, Opie-Martin S, et al. ALSgeneScanner: a pipeline for the analysis and interpretation of DNA sequencing data of ALS patients. Amyotroph Lateral Scler Frontotemporal Degener. 2019;20(3-4):207-15.

25. Condello C, Ayers JI, Dalgard CL, Garcia Garcia MM, Rivera BM, Seeley WW, et al. Guam ALS-PDC is a distinct double-prion disorder featuring both tau and Abeta prions. Proc Natl Acad Sci U S A. 2023;120(13):e2220984120.

26. Tate JG, Bamford S, Jubb HC, Sondka Z, Beare DM, Bindal N, et al. COSMIC: the Catalogue Of Somatic Mutations In Cancer. Nucleic Acids Res. 2019;47(D1):D941-D7.

27. Abascal F, Harvey LMR, Mitchell E, Lawson ARJ, Lensing SV, Ellis P, et al. Somatic mutation landscapes at single-molecule resolution. 2021;593(7859):405-10.

28. Keren-Shaul H, Spinrad A, Weiner A, Matcovitch-Natan O, Dvir-Szternfeld R, Ulland TK, et al. A Unique Microglia Type Associated with Restricting Development of Alzheimer's Disease. Cell. 2017;169(7):1276-90 e17.
29. Deczkowska A, Keren-Shaul H, Weiner A, Colonna M, Schwartz M, Amit I. Disease-Associated Microglia: A Universal Immune Sensor of Neurodegeneration. Cell. 2018;173(5):1073-81.
30. Schwab C, Steele JC, McGeer PL. Neurofibrillary tangles of Guam parkinson-dementia are associated with reactive microglia and complement proteins. Brain Res. 1996;707(2):196-205.
31. Majerova P, Garruto RM, Kovac A. Cerebrovascular inflammation is associated with tau pathology in Guam parkinsonism dementia. J Neural Transm (Vienna). 2018;125(7):1013-25.
32. Mills JD, Kavanagh T, Kim WS, Chen BJ, Waters PD, Halliday GM, et al. High expression of long intervening non-coding RNA OLMALINC in the human cortical white matter is associated with regulation of oligodendrocyte maturation. Mol Brain. 2015;8:2.
33. Morimoto S, Ishikawa M, Watanabe H, Isoda M, Takao M, Nakamura S, et al. Brain Transcriptome Analysis Links Deficiencies of Stress-Responsive Proteins to the Pathomechanism of Kii ALS/PDC. Antioxidants (Basel). 2020;9(5).
34. Anderson FH, Richardson EP, Jr., Okazaki H, Brody JA. Neurofibrillary degeneration on Guam: frequency in Chamorros and non Chamorros with no known neurological disease. Brain. 1979;102(1):65-77.
35. Perl DP, Hof PR, Purohit DP, Loerzel AJ, Kakulas BA. Hippocampal and entorhinal cortex neurofibrillary tangle formation in Guamanian Chamorros free of overt neurologic dysfunction. J Neuropathol Exp Neurol. 2003;62(4):381-8.
36. Verheijen BM, Lussier C, Muller-Hubers C, Garruto RM, Oyanagi K, Braun RJ, et al. Activation of the Unfolded Protein Response and Proteostasis Disturbance in Parkinsonism-Dementia of Guam. J Neuropathol Exp Neurol. 2020;79(1):34-45.
37. Dolzhenko E, van Vugt J, Shaw RJ, Bekritsky MA, van Blitterswijk M, Narzisi G, et al. Detection of long repeat expansions from PCR-free whole-genome sequence data. Genome Res. 2017;27(11):1895-903.
38. Youk J, Kwon HW, Lim J, Kim E, Kim R, Park S, et al. Mutational impact and signature of ionizing radiation. Biorxiv. 2021.
39. Dobin A, Davis CA, Schlesinger F, Drenkow J, Zaleski C, Jha S, et al. STAR: ultrafast universal RNA-seq aligner. Bioinformatics. 2013;29(1):15-21.
40. Anders S, Pyl PT, Huber W. HTSeq--a Python framework to work with high-throughput sequencing data. Bioinformatics. 2015;31(2):166-9.
41. Love MI, Huber W, Anders S. Moderated estimation of fold change and dispersion for RNA-seq data with DESeq2. Genome Biol. 2014;15(12):550.
