# Supplementary material for: The cycad genotoxin methylazoxymethanol, linked to Guam ALS/PDC, induces transcriptional mutagenesis
Source: Acta Neuropathol Commun. 2024 Feb 21;12:30. doi: 10.1186/s40478-024-01725-y (PMC10882831; doi:10.1186/s40478-024-01725-y)
Supplement: Supplementary file 1 — Additional file 1. Supplementary material 1 (ZIP 1015 kb) [file 40478_2024_1725_MOESM1_ESM.zip › Additional File 1/Supplementary Table 3. Demographic and genetic findings.pdf]

Supplementary Table 3. Demographic and genetic findings for Guam ALS/PDC and control cases

Group	ID	Sex	Race	Age at onset	Age at death	*C9ORF72* repeats	*ATXN2* repeats	*APOE* genotype	*MAPT*-related risk SNP		
									rs242557	rs2258689	rs242944
ALS/PDC	05335	M	Chamorro	63	69	2/6	22/23	ε3/ε3	G/A	-/-	-/-
ALS/PDC	05317	M	Chamorro	37	60	6/12	22/22	ε3/ε3	G/A	T/T	-/-
ALS/PDC	05319	M	Chamorro	52	54	2/6	22/22	ε3/ε3	G/A	T/T	G/G
ALS/PDC	05324	M	Chamorro	46	51	2/5	22/22	ε3/ε4	G/G	T/T	-/-
ALS/PDC	05328	M	Chamorro	44	49	2/8	22/22	ε3/ε3	G/A	T/T	G/G
ALS/PDC	05336	M	Chamorro	42	45	2/6	22/22	ε3/ε3	A/A	-/-	G/G
ALS/PDC	05312	F	Chamorro	66	69	2/6	22/22	ε3/ε4	G/A	-/-	G/G
ALS/PDC	05313	M	Chamorro	59	61	2/11	22/22	ε3/ε3	G/G	T/T	-/-
ALS/PDC	05314	F	Chamorro	47	52	2/8	22/22	ε3/ε3	G/A	T/T	G/G
ALS/PDC	05316	M	Chamorro	42	52	2/8	22/22	ε3/ε3	A/A	-/-	-/-
ALS/PDC	05320	F	Chamorro	59	64	6/8	22/22	ε3/ε3	A/A	-/-	G/G
ALS/PDC	05321	M	Chamorro	61	73	5/8	22/22	ε3/ε3	A/A	-/-	G/G
ALS/PDC	05323	F	Chamorro	46	51	12/17	22/23	ε3/ε3	A/A	-/-	G/G
ALS/PDC	05334	M	Chamorro	61	76	2/12	22/22	ε3/ε3	A/A	T/T	G/G

HC	05318	M	Chamorro	-	72	2/6	22/22	ε3/ε3	G/A	T/T	G/G
HC	05322	F	Chamorro	-	73	7/16	22/22	ε3/ε3	G/G	T/T	G/G
HC	05325	F	Chamorro	-	68	2/14	22/22	ε3/ε3	G/A	-/-	-/-
HC	05326	F	Chamorro	-	57	2/2	22/22	ε3/ε3	A/A	-/-	G/G
HC	05327	M	Chamorro	-	48	12/12	22/22	ε3/ε3	A/A	T/T	G/G
HC	05330	M	Chamorro	-	27	6/8	22/22	ε2/ε3	A/A	-/-	G/G
HC	05331	F	Chamorro	-	43	2/8	19/22	ε3/ε3	G/A	T/T	-/-
HC	05332	M	Chamorro	-	63	2/6	22/22	ε3/ε3	G/G	-/-	-/-
HC	05337	M	Chamorro	-	56	2/11	22/22	ε3/ε3	G/A	-/-	G/G

ALS/PDC: amyotrophic lateral sclerosis/parkinsonism-dementia complex, *APOE*: apolipoprotein E, *ATXN2*: ataxin-2, *C9ORF72*: chromosome 9 open reading frame 72, HC: healthy control, *MAPT*: microtubule-associated protein tau
