# Supplementary material for: The cycad genotoxin methylazoxymethanol, linked to Guam ALS/PDC, induces transcriptional mutagenesis
Source: Acta Neuropathol Commun. 2024 Feb 21;12:30. doi: 10.1186/s40478-024-01725-y (PMC10882831; doi:10.1186/s40478-024-01725-y)
Supplement: Supplementary file 1 — Additional file 1. Supplementary material 1 (ZIP 1015 kb) [file 40478_2024_1725_MOESM1_ESM.zip › Additional File 1/Supplementary Table 5. Genome-wide assessment of variants.pdf]

Supplementary Table 5. Genome-wide assessment of Guam ALS/PDC and control cases for ALS genetic risk variants

Group	ID	Gene	Variants	Result	Homozygosity	CADD	MAF	Classification
ALS/PDC	05336	*VWA8*	c.1408C>T	p.Pro470Ser	heterozygous	24.5	-	VUS
ALS/PDC	05320	*NEFH*	c.1947_1964dup	inframe insertion	heterozygous	-	-	Benign
HC	05318	*PLCG2*	c.2173C>T	p.Leu725Phe	homozygous	23.8	-	VUS
HC	05318	*NEFH*	c.2232_2249del	inframe deletion	heterozygous	-	-	Benign
HC	05330	*FUS*	c.669CGG	inframe insertion	heterozygous	-	-	Benign

ALS/PDC: amyotrophic lateral sclerosis/parkinsonism-dementia complex, CADD: combined annotation dependent depletion, HC: healthy control, MAF: minor allele frequency, VUS: variant of undetermined significance
