# Supplementary material for: The cycad genotoxin methylazoxymethanol, linked to Guam ALS/PDC, induces transcriptional mutagenesis
Source: Acta Neuropathol Commun. 2024 Feb 21;12:30. doi: 10.1186/s40478-024-01725-y (PMC10882831; doi:10.1186/s40478-024-01725-y)
Supplement: Supplementary file 2 — Additional file 2. Supplementary material 2 (ZIP 444 kb) [file 40478_2024_1725_MOESM2_ESM.zip › Additional File 2/Supplementary Information 2.pdf]

Supplementary Information 2

Transcriptional mutagenesis experiments with methylazoxymethanol (MAM)

Bert M. Verheijen, Claire Chung, Ben Thompson, Hyunjin Kim, Jasper J. Anink, Asa Nakahara, James D. Mills, NYGC ALS consortium, Jeong H. Lee, Eleonora Aronica, Kiyomitsu Oyanagi, Akiyoshi Kakita, Jean-Francois Gout, Marc Vermulst

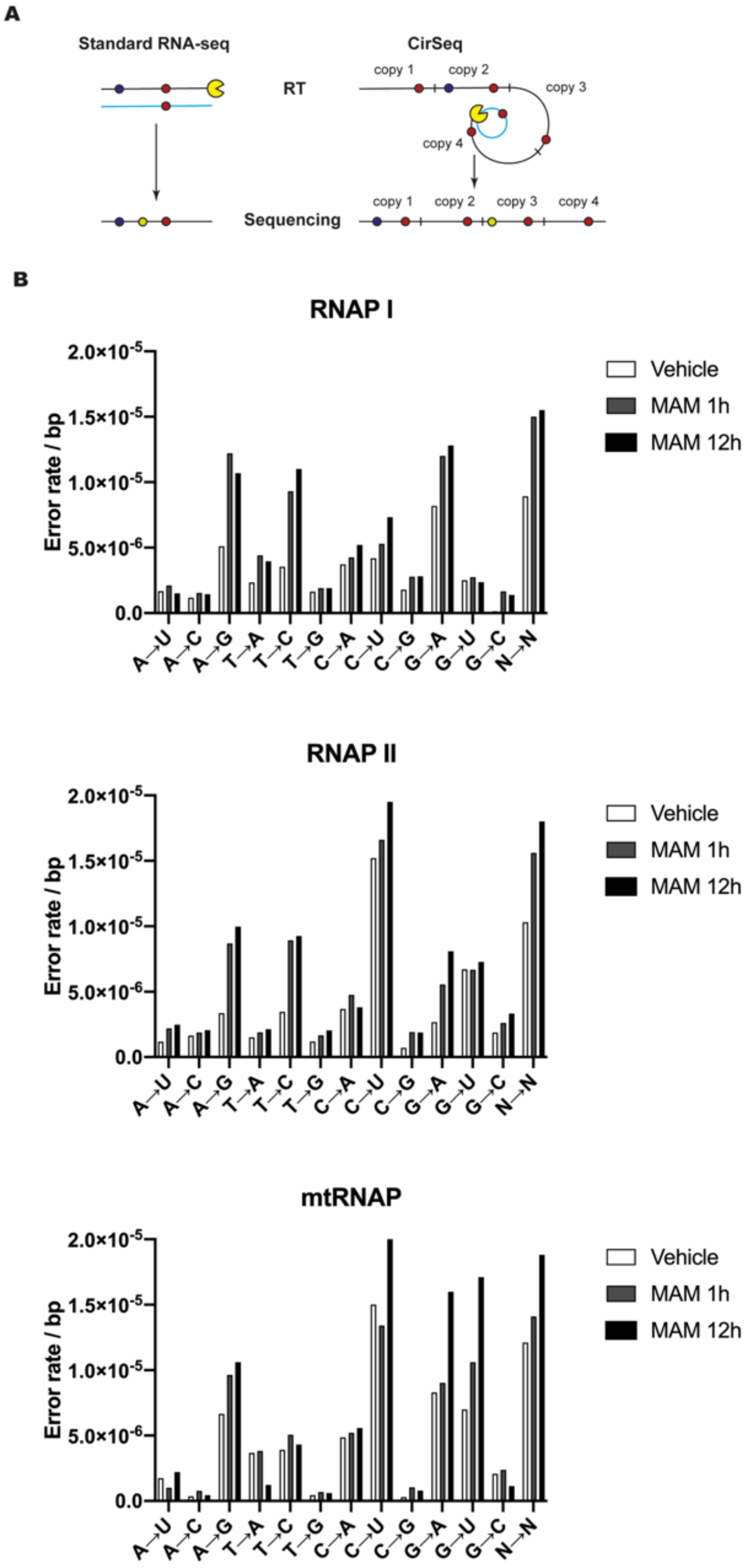
A
Standard RNA-seq
CirSeq
RT
copy 1
copy 2
copy 3
copy 4
Sequencing
B
RNAP I
RNAP II
mtRNAP
Error rate / bp
2.0×10^-5
1.5×10^-5
1.0×10^-5
5.0×10^-6
0.0
A→U
A→C
A→G
T→A
T→C
T→G
C→A
C→U
C→G
G→A
G→U
G→C
N→N
Vehicle
MAM 1h
MAM 12h

Sup. Fig. 3. Methylazoxymethanol (MAM) acetate exposure induces transcriptional mutagenesis in mouse primary fibroblasts. Adult mouse ear pinnae-derived primary fibroblasts were contact-inhibited by growing them at confluency for 3 days. Next, arrested cells were treated with 1 mM methylazoxymethanol (MAM) acetate (MRI Global) or vehicle (PBS) for 1h, rinsed with PBS three times, and incubated in culturing medium for 1h or 12h. RNA was then extracted and processed for rolling-circle consensus sequencing (CirSeq) as described previously (1, 2). (A) CirSeq entails circularization of single-stranded RNA fragments (i.e., it generates RNAs with a closed loop structure) followed by rolling-circle reverse transcription (RT) with random primers. RT of circular RNA templates results in the generation of concatemerized cDNA fragments, which can subsequently be used for library preparation and sequencing. The tandem repeats present in the sequences can be used for consensus-based correction of errors generated during library preparation (e.g., errors introduced during RT) and sequencing. Genuine transcription errors are indicated by red dots, artifacts with other colors. (B) CirSeq data showed that MAM induces an overall increase in transcription errors in fibroblasts (N→N), including a small increase in C→U errors committed by RNAPII (source data in **Supplementary Table 8**). Note that the CirSeq data cannot be directly compared to the single-cell RNA-sequencing data described in the present study. These sequencing methods are fundamentally different and the CirSeq assay has several limitations, e.g., it cannot distinguish direct damage to RNA molecules from genuine transcription errors. In addition, differences between cell types (fibroblasts vs. NSCs) and associated culturing conditions as well as different incubation times following MAM treatment could account for different results. Lastly, contact inhibition may not have resulted in complete cell cycle arrest and still replicating cells in these cultures could, for example, have introduced DNA mutations that confound the CirSeq dataset.

Methods

Single-cell transcriptional mutagenesis experiments with methylazoxymethanol (MAM)

For single-cell sequencing experiments, mouse NSCs were cultured as described previously (3). Cell cycle arrest was confirmed by Ki-67 staining as described in (4). Quiescent NSCs were treated with 1mM MAM acetate (MRI Global Chemical Carcinogen Repository) (n=2) or vehicle (PBS) (n=2) for 1h, after which they were rinsed with PBS three times and allowed to recover in quiescence medium for 16h. MAM dose was based on the maximal dose that did not induce significant apoptotic cell death as determined by terminal deoxynucleotidyl transferase (TdT) dUTP Nick-End Labeling (TUNEL) assays. All experiments were performed using a single MAM stock (toxicity and dose should be determined for separately prepared stocks of MAM). Cells were detached by scraping and a fraction of the detached cells were counted by flow cytometry (MACSQuant Analyzer 10, Miltenyi Biotec). Remaining cells were then processed using the 10x Genomics workflow (10× Genomics Chromium Single-Cell Reagent Kit [v3.1 Single Index]; 10x Genomics Chromium controller). All samples were processed directly to avoid freeze-thaw cycles. Analysis of transcript errors was performed as described previously (4). Transcript error rates can be found in **Supplementary Table 9** and data on pseudo-alleles is provided in **Supplementary Table 10**.

References

1. Fritsch C, Gout JP, Vermulst M. Genome-wide Surveillance of Transcription Errors in Eukaryotic Organisms. J Vis Exp. 2018(139).
2. Fritsch C, Gout JF, Haroon S, Towheed A, Chung C, LaGosh J, et al. Genome-wide surveillance of transcription errors in response to genotoxic stress. Proc Natl Acad Sci U S A. 2021;118(1).
3. Morrow CS, Porter TJ, Xu N, Arndt ZP, Ako-Asare K, Heo HJ, et al. Vimentin Coordinates Protein Turnover at the Aggresome during Neural Stem Cell Quiescence Exit. Cell Stem Cell. 2020;26(4):558-68 e9.
4. Chung C, Kou Y, Shemtov S, Verheijen BM, Flores I, Love K, et al. Transcript errors generate a continuous stream of amyloid and prion-like proteins in human cells. Biorxiv. 2023.
